# H-Ferritin is essential for macrophages’ capacity to store or detoxify exogenously added iron

**DOI:** 10.1038/s41598-020-59898-0

**Published:** 2020-02-20

**Authors:** Gonçalo Mesquita, Tânia Silva, Ana C. Gomes, Pedro F. Oliveira, Marco G. Alves, Rui Fernandes, Agostinho A. Almeida, Ana C. Moreira, Maria Salomé Gomes

**Affiliations:** 1 i3S – Instituto de Investigação e Inovação em Saúde, Universidade do Porto, Porto, Portugal; 2 IBMC – Instituto de Biologia Molecular e Celular, Universidade do Porto, Porto, Portugal; 3 Departamento de Biologia Molecular, ICBAS – Instituto de Ciências Biomédicas Abel Salazar, Universidade do Porto, Porto, Portugal; 4 Department of Microscopy, Laboratory of Cell Biology and Unit for Multidisciplinary Research in Biomedicine (UMIB), ICBAS, Universidade do Porto, Porto, Portugal; 5 Department of Genetics, Faculty of Medicine (FMUP), Universidade do Porto, Porto, Portugal; 6 LAQV/REQUIMTE, Departamento de Ciências Químicas, Faculdade de Farmácia, Universidade do Porto, Porto, Portugal

**Keywords:** Biochemistry, Cell biology, Immunology

## Abstract

Macrophages are central cells both in the immune response and in iron homeostasis. Iron is both essential and potentially toxic. Therefore, iron acquisition, transport, storage, and release are tightly regulated, by several important proteins. Cytosolic ferritin is an iron storage protein composed of 24 subunits of either the L- or the H-type chains. H-ferritin differs from L-ferritin in the capacity to oxidize Fe^2+^ to Fe^3+^. In this work, we investigated the role played by H-ferritin in the macrophages’ ability to respond to immune stimuli and to deal with exogenously added iron. We used mice with a conditional deletion of the H-ferritin gene in the myeloid lineage to obtain bone marrow-derived macrophages. These macrophages had normal viability and gene expression under basal culture conditions. However, when treated with interferon-gamma and lipopolysaccharide they had a lower activation of Nitric Oxide Synthase 2. Furthermore, H-ferritin-deficient macrophages had a higher sensitivity to iron-induced toxicity. This sensitivity was associated with a lower intracellular iron accumulation but a higher production of reactive oxygen species. These data indicate that H-ferritin modulates macrophage response to immune stimuli and that it plays an essential role in protection against iron-induced oxidative stress and cell death.

## Introduction

Iron is essential for almost all living organisms, due to its key roles in biological processes such as DNA and RNA synthesis, mitochondrial respiratory chain, cell proliferation, and differentiation, among others^1^. On the other hand, iron needs to be strictly controlled and stored, due to its potential to cause cell damage^2^. Proteins like ferritin, transferrin, and ferroportin are essential to control the intracellular and intercellular iron fluxes and imbalances in their expression or function lead to severe health problems^3–5^. In particular, ferritin is essential to safely store iron inside cells, avoiding its harmful effects. This protein is composed of a 24 subunits complex that forms a nanocage, inside which iron is trapped^6^. Ferritin subunits can be of the H, L (cytosol) or M (mitochondria) types^7^. The cytosolic subunits have distinct functions and are present in different proportions in different cell types^8^.

H-ferritin (FTH1) is essential for mammalians’ development, as the full knock-out of its gene leads to embryonic death in mice^9^. FTH1 differs from L-ferritin (FTL) in its ferroxidase activity, which is responsible for the oxidation of iron Fe^2+^ to Fe^3+^, preventing the oxidative damage that could be caused by Fenton reactions involving Fe^2+^ ^10^. These reactions can generate oxygen reactive species that damage proteins, lipids, and other cell components.

Macrophages are central players in iron metabolism. They recycle senescent red blood cells and nurse the formation of new ones^11–13^, thus participating in the major systemic iron fluxes occurring in higher vertebrates. Macrophages are also a first line of defence against pathogens, and they modulate iron availability as part of host protective mechanisms^12,14–16^.

Since macrophages and iron metabolism are tightly connected and H-ferritin is crucial for iron storage inside the cells, we hypothesized that H-ferritin has a key role in macrophages viability, development, activation, and iron handling. To test this hypothesis, we used mice with a conditional deletion of the H-ferritin gene (*Fth1*) in the myeloid lineage, generated from the H-ferritin-loxP mice created by Lukas Kuhn and collaborators^17^. We obtained bone marrow-derived macrophages (BMDM) from these mice and found that although *in vitro* macrophage differentiation proceeded normally, H-ferritin-deficient BMDM had subtle alterations in their response to immune stimulation and a marked increase in susceptibility to oxidative stress and cell death induced by exogenously added iron.

## Results

### H-ferritin is not necessary for ***in vitro*** differentiation of bone marrow-derived macrophages

**Figure 1:**
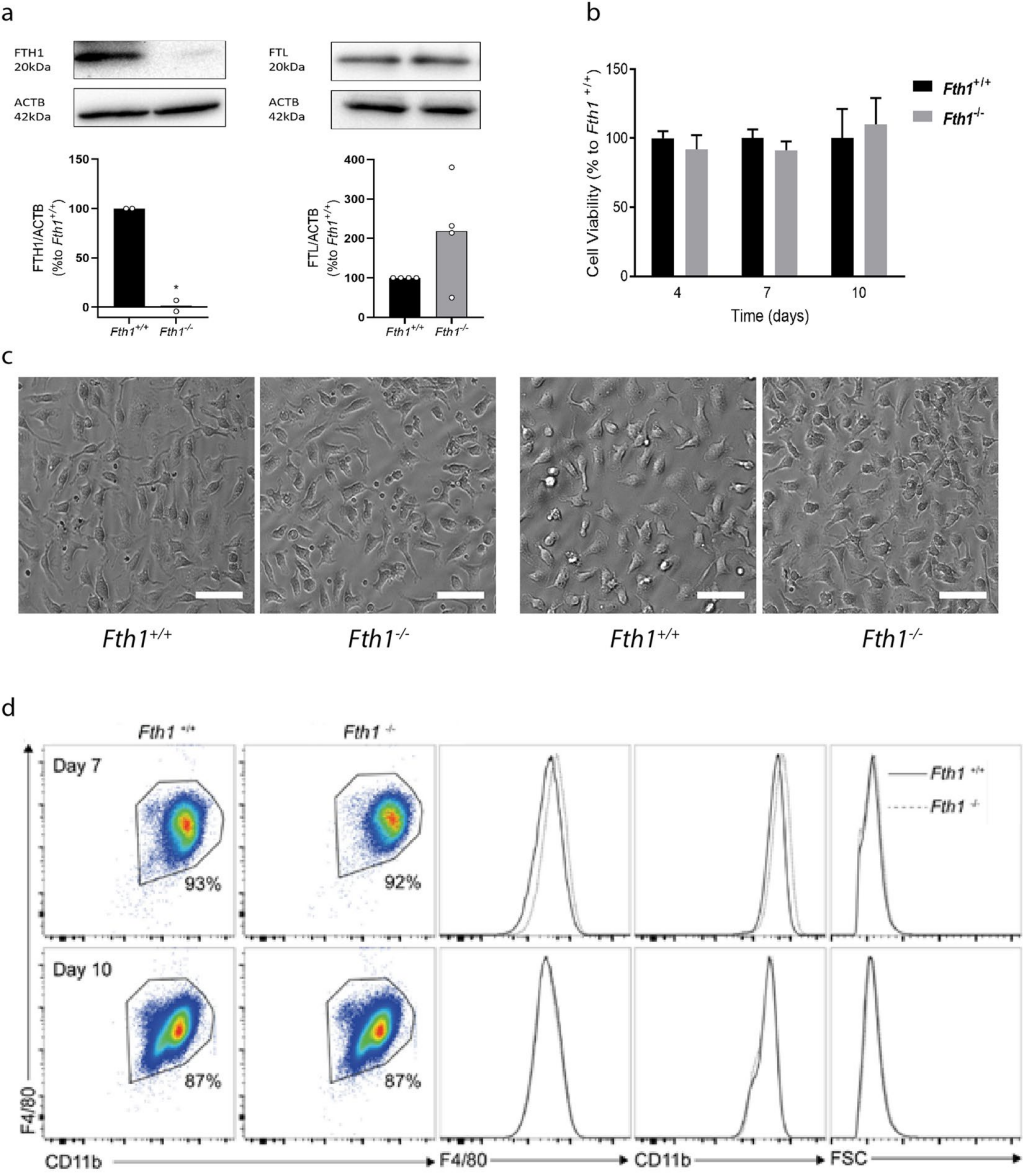
H-ferritin is not necessary for *in vitro* differentiation of bone marrow-derived macrophages. (**a**) Quantification of FTH1 and FTL by Western blot in protein extracts from *Fth1*^+/+^ (black) and *Fth1*^−/−^ (grey) BMDM, at 10 days of culture. Quantification was made by densitometry analysis with ImageLab^TM^ software of FTH1 or FTL bands normalized to β-actin (ACTB) in samples from 2–4 independent cultures and are represented as percentage relative to *Fth1*^+/+^ BMDM (t test *p < 0.05). (**b**) Cell viability of *Fth1*^+/+^ (black) and *Fth1*^−/−^ (grey) BMDM was measured at 4, 7 and 10 days of culture, by resazurin reduction. The results represent the mean + SD of at least three independent cultures. The graphs depict the percentage of *Fth1*^−/−^ viable cells relative to *Fth1*^+/+^ cultured in parallel. (**c**) Light microscopy images of BMDM at 7 (left panels) and 10 (right panels) days of culture. The cells were visualized and imaged in a Leica DMI6000 Time-lapse microscope. Images are representative of the cultures obtained from four animals per genotype. Scale bar: 50 μm. (**d**) Flow cytometry analysis of BMDM at 7 and 10 days of differentiation. Cells were stained for the myeloid markers F4/80 and CD11b. CD11b^+^ F4/80^+^ cells were considered completely differentiated macrophages. Left panels: flow cytometry plots gated for CD11b^+^ F4/80^+^ cells. Right panels: histogram plots of *Fth1*^+/+^ (solid black line) and *Fth1*^−/−^ (dotted grey line) for F4/80, CD11b, and cell size (FSC).

Since H-ferritin is essential for mouse development^9^, we started by evaluating whether it was also necessary for *in vitro* differentiation of macrophages from their bone marrow (BM) precursors. Bone marrow-derived macrophages (BMDM) were obtained from *Fth1*^*Fl/Fl*^*; Lyz2*^+/+^ (*Fth1*^+/+^, H-ferritin-sufficient) and *Fth1*^*Fl/Fl*^*; Lyz2*^*cre/+*^ (*Fth1*^−/−^, H-ferritin-deficient) mice. The absence of H-ferritin in *Fth1*^−/−^ BMDM was confirmed by western-blot (Fig. 1a) and also by gene expression (Table 1). Although it tended to be increased, the level of L-ferritin was not significantly different between *Fth1*^−/−^ and *Fth1*^+/+^ macrophages, either at the protein (Fig. 1a) or at the RNA level (Table 1). Throughout the culture, cells with either genotype were indistinguishable in terms of morphology, as well as in terms of viability (measured by a resazurin reduction assay) and in the acquisition of macrophage differentiation markers (evaluated by flow cytometry) (Fig. 1b–d). These data also showed that for both genotypes, at the 7^th^ and 10^th^ day of culture, nearly 90% of the cells had acquired the macrophage phenotypic markers (Fig. 1d). Thus, subsequent experiments were performed 7 days after the beginning of the culture.

**Table 1:** Basal gene expression in *Fth1*^−/−^ BMDM as compared to *Fth1*^+/+^ cells.

Gene of interest	Basal Level^a^
*Fth1*	0.02 ± 0.02*
*Ftl*	0.69 ± 0.35
*Tfrc*	1.39 ± 1.11
*Slc40a1*	1.16 ± 1.08
*Hmox1*	0.60 ± 0.33
*Nos2a*	0.83 ± 1.19
*Arg1*	0.87 ± 1.19
*Tnf*	0.63 ± 0.34

^a^The values were calculated as the fold change relative to *Fth1*^+/+^ cells, and represent the mean ± SD of four to eleven independent cultures per gene. Statistical analysis was performed using multiple t-test *p < 0.05.

In addition, we asked whether the absence of H-ferritin expression would impact the basal iron and inflammatory status of the macrophages. For that, we measured the expression of genes involved in the cellular handling of iron: transferrin receptor (*Tfrc*, iron uptake), ferroportin (*Slc40a1*, iron export), heme oxygenase-1 (*Hmox1*, heme degradation), inducible nitric oxide synthase (*Nos2a*), arginase (*Arg1*) and tumor necrosis factor (*Tnf*) (macrophage activation), and we did not find any significant differences between the two genotypes (Table 1).

### H-ferritin modulates macrophage response to immune activation

**Figure 2:**
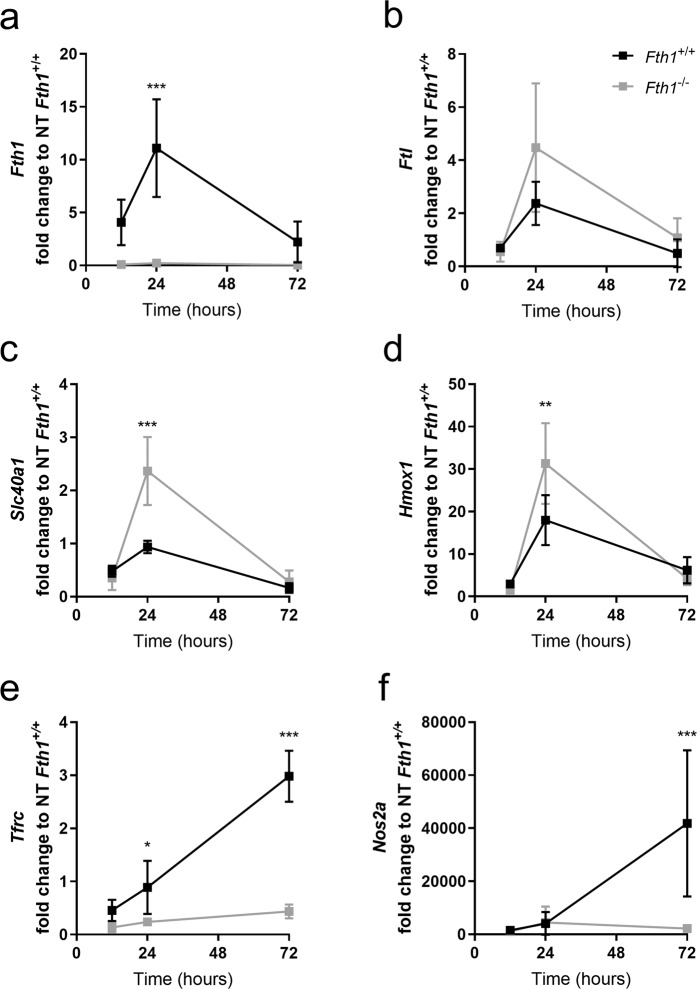
Gene expression is altered in *Fth1*^+/+^ and *Fth1*^−/−^ BMDM upon treated with IFNG plus LPS. *Fth1*^+/+^ (black) and *Fth1*^−/−^ (grey) BMDM were treated with IFNG plus LPS, for 12, 24 or 72 hours. Gene expression for the genes of interest **(a)** *Fth1*, **(b)** *Ftl*, **(c)** *Slc40a1*, **(d)** *Hmox1*, **(e)** *Tfrc* and **(f)** *Nos2a* were normalized to the level of *Hprt1*. Data are shown as fold change (mean ± SD) relative to the non-treated *Fth1*^+/+^ cells, calculated with the 2^−ΔΔCT^ method, of at least three independent experiments. Statistical analysis was obtained by two-way ANOVA, *p < 0.05, **p < 0.01 and ***p < 0.001.

In order to investigate the potential role of H-ferritin in macrophage activation, interferon gamma and lipopolysaccharide (IFNG + LPS) were added to the BMDM on the 7^th^ day of culture, as described in Materials and Methods. No differences in cell viability were observed during the treatment with IFNG + LPS in cells of either genotype (data not shown). The effects of treatment on macrophage gene expression were evaluated 12, 24 and 72 hours later (Fig. 2 and Table S1). In wild-type cells, *Fth1* gene expression increased upon treatment with IFNG + LPS, especially at 24 h (Fig. 2a), in accordance with previous reports^14^. *Ftl* expression slightly increases with IFNG + LPS treatment at 24 h, however, no differences were observed between the two genotypes (Fig. 2b). Interestingly, the levels of *Slc40a1*, which codes for the iron exporter ferroportin, increased during the first 24 h of treatment and decreased at 72 h (Fig. 2c). Of note, at 24 h post-treatment, the expression of *Slc40a1* was significantly higher in *Fth1*^−/−^ macrophages than in wild-type cells. The expression of *Hmox1* also increased with treatment, with *Fth1*^−/−^ cells having a significantly higher expression than wild-type cells at 24 h post-treatment (Fig. 2d). The expression of *Tfrc* in *Fth1*^+/+^ cells increased with time, whereas in *Fth1*^−/−^ cells its expression was significantly lower at all the evaluated time-points (Fig. 2e). The gene showing the highest induction with treatment was *Nos2a*, a hallmark of macrophage activation^18^ (Fig. 2f). However, the induction of *Nos2a* upon treatment with IFNG + LPS was significantly lower in *Fth1*^−/−^ macrophages when compared to *Fth1*^+/+^ cells (Fig. 2f).

**Figure 3:**
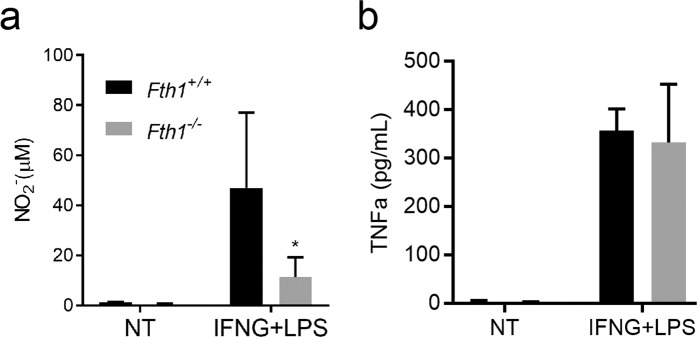
*Fth1*^−/−^ macrophages have decreased production of nitric oxide but normal release of TNFa as compared to *Fth1*^+/+^ macrophages. *Fth1*^+/+^ (black) and *Fth1*^−/−^ (grey) BMDM were treated with IFNG plus LPS, for 3 days. (**a**) Nitrite production was quantified in the supernatant by the Griess assay. (**b**) The release of TNFa from BMDM was measured by ELISA. The results shown represent the mean + SD of two to six independent cultures per group (two-way ANOVA with Tukey multiple comparisons test; *p < 0.05).

Macrophage activation was also evaluated by measuring the release of nitrites and tumor necrosis factor alfa (TNFa) to the culture supernatants. In agreement with the *Nos2a* gene expression data, significantly lower levels of nitrites were found in the supernatants of *Fth1*^−/−^ than in *Fth1*^+/+^ macrophage cultures treated with IFNG + LPS (Fig. 3a). However, the release of TNFa was the same, independently of the expression of *Fth1* (Fig. 3b).

### H-ferritin supports macrophage survival in the presence of exogenous iron

**Figure 4:**
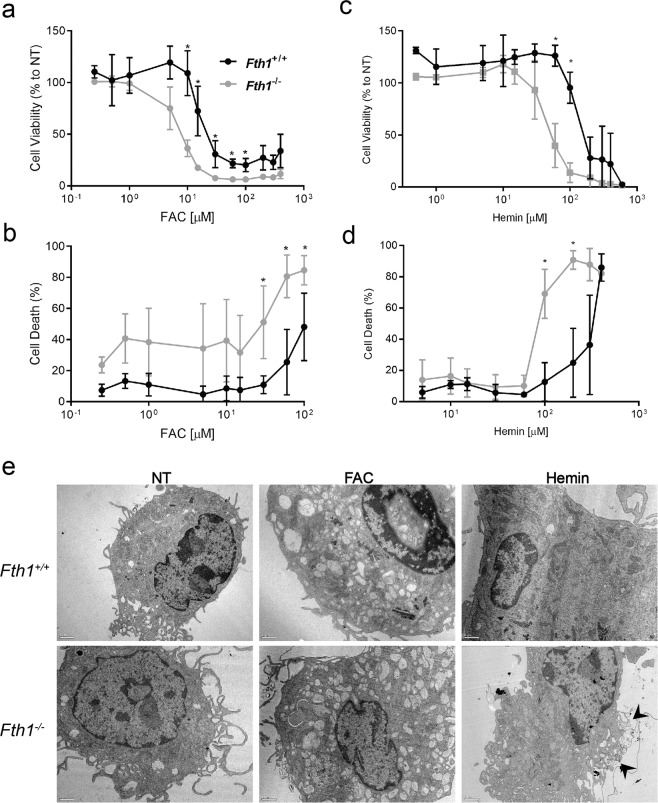
H-ferritin supports macrophage survival in the presence of exogenous iron. *Fth1*^+/+^ (black) and *Fth1*^−/−^ (grey) BMDM were treated with increasing concentrations of (**a,b**) FAC or (**c,d**) hemin, for 3 days. (**a,c**) Cell viability was measured by resazurin reduction. The results represent the mean ± SD of three to seven independent cultures per condition and are expressed as the percentage of viable cells relative to non-treated cells of each genotype. (**b,d**) Cell death was measured through SYTOX Green incorporation into dead cells. The results represent the mean ± SD of three to seven independent cultures per condition (two-way ANOVA with Tukey multiple comparisons test; *p < 0.05). (**e**) Transmission electron microscopy images of *Fth1*^+/+^ (upper panels) and *Fth1*^−/−^ (lower panels) BMDM treated with 10 μM of FAC or 100 μM of hemin for 12 h. Scale bar: 1 μm. The images are representative of three independent cultures per condition. Arrowheads: membrane damage.

Although *Fth1*^−/−^ macrophages had normal basal iron-related gene expression (Table 1), we hypothesized that these cells could have a hampered capacity to deal with exogenously added iron. To assess that, cells were treated with different concentrations of ferric ammonium citrate (FAC) or hemin. Cellular viability was evaluated by resazurin reduction 3 days after treatment. As expected, FAC and hemin caused a concentration-dependent reduction of cell viability, but more interestingly, *Fth1*^−/−^ macrophages were more susceptible to iron toxicity than *Fth1*^+/+^ macrophages (Fig. 4a,c and Table 2). Moreover, FAC was more toxic than hemin, for both cell types (Table 2).

**Table 2:** Relative toxicity of FAC and hemin for *Fth1*^+/+^ and *Fth1*^−/−^ BMDM.

Compound	IC_50_ ± SD (µM)^a^	
	*Fth1*^+/+^	*Fth1*^−/−^
FAC	26.6 ± 1.2	9.7 ± 1.1
Hemin	150.9 ± 1.1	49.6 ± 1.0

^a^Each value represents the average ± SD of three to seven independent cultures per condition. IC_50_ is the concentration that inhibits 50% of macrophage viability after 3 days of incubation with each compound.

In order to confirm these results, we evaluated the impact of the same compounds in macrophage cell death using the membrane-impermeable dye SYTOX Green^TM^. Both FAC and hemin caused a dose-dependent increase in cell death, which was again more marked in *Fth1*^−/−^ cells compared to *Fth1*^+/+^ cells (Fig. 4b,d). The damage induced by exogenous iron was also evident in morphological alterations detected by Transmission Electron Microscopy (TEM) (Fig. 4e). In cells treated with FAC, the main alteration observed was an increase in the number of vesicles in the cytoplasm. Some cells also had nuclear alterations suggestive of necrosis. With the hemin treatment, cells were larger and there were signs of membrane damage (Fig. 4e, arrows). These alterations were more frequent and marked in *Fth1*^−/−^ cells (Fig. 4e).

The data presented here indicate that *Fth1*^−/−^ cells are less capable of handling exogenous iron, being more sensitive to iron-induced cell death.

### H-ferritin-deficient macrophages retain less exogenously added iron

**Figure 5:**
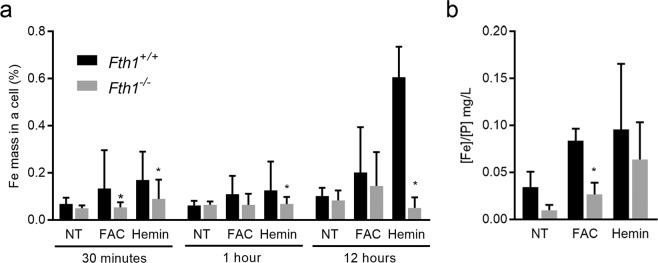
H-ferritin-deficient macrophages retain less exogenously added iron. Iron was quantified in *Fth1*^+/+^ (black) and *Fth1*^−/−^ (grey) BMDM treated with 10 μM of FAC or 100 μM of hemin. (**a**) Iron quantification by TEM-EDS at 30 minutes, 1 or 12 hours, post-treatments. The results represent the mean + SD of 6 to 24 cells and are expressed as the percentage of iron mass inside a single cell (two-way ANOVA with Tukey multiple comparisons test; *p < 0.05). (**b**) Iron quantification by atomic absorption at 24 h post-treatments. The results represent the mean + SD of 4 independent cultures per condition and are expressed as the ratio between the concentration of iron and the concentration of phosphorus in each sample (two-way ANOVA with Tukey multiple comparisons test; *p < 0.05).

In order to evaluate how H-ferritin deficiency impacted iron internalization and retention by macrophages, we used TEM equipped with an Energy-Dispersive X-ray Spectroscopy (EDS) system to trace intracellular iron. Cells were analysed 30 minutes, 1 and 12 hours after iron treatments. In all the time-points, a clear tendency was observed for lower intracellular iron levels in *Fth1*^−/−^ as compared to *Fth1*^+/+^ cells (Fig. 5a). This difference was statistically significant at all time-points with hemin, but only at 30 minutes post-treatment with FAC (Fig. 5a). We complemented this assessment by measuring intracellular iron levels 24 hours after iron addition, using atomic absorption spectrometry. We found that *Fth1*^−/−^ BMDM had a significantly lower amount of intracellular iron than *Fth1*^+/+^ cells after treatment with FAC (Fig. 5b). Overall, these results indicate that the lack of H-ferritin hampers macrophages’ capacity to retain iron when this is exogenously added.

**Figure 6:**
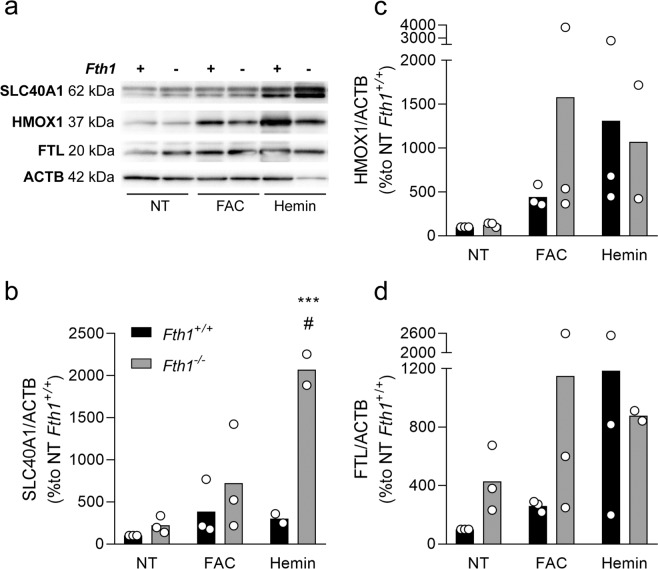
H-ferritin-deficient macrophages express higher ferroportin protein levels upon hemin treatment. Quantification of SLC40A1, HMOX1 and FTL by Western blot in protein extracts from *Fth1*^+/+^ (black) and *Fth1*^−/−^ (grey) BMDM, 24 hours after treatment with 10 µM of FAC or 100 µM of hemin. **(a)** Representative image of the quantification of each protein by western blot. **(b–d)** Quantification of SLC40A1 (**b**), HMOX1 (**c**) and FTL (**d**), made by densitometry analysis with ImageLab^TM^ software. The bands were normalized to ACTB, and the results are presented as percentage relative to the non-treated *Fth1*^+/+^ cells, average ± standard deviation from 3 independent experiments. Statistical analysis was performed using two-way ANOVA with Tukey’s multiple comparisons test. ***p < 0.001 when compared with *Fth1*^+/+^, ^#^< 0.05 when compared with non-treated *Fth1*^−/−^.

In order to understand the mechanisms underlying these differences in cellular iron dynamics, we evaluated the alterations in gene expression occurring upon iron treatments (Tables 3 and S2). In general, *Fth1*^−/−^ macrophages exhibited similar trends as *Fth1*^+/+^ macrophages in the response to the iron treatments, with upregulation of genes involved in iron storage (*Ftl*) and iron export (*Slc40a1*) as well as with a decrease in the expression of the iron internalization-related gene *Tfrc* in response to heme (Table 3). However, a tendency was observed for an increased expression of *Slc40a1* in *Fth1*^−/−^ in comparison with wild-type cells. This tendency was also observed at the protein level, although it only reached statistical significance in the case of the hemin treatment (Fig. 6b). We did not observe statistically significant differences between *Fth1*^+/+^ and *Fth1*^−/−^ macrophages concerning the protein expression levels of FTL (Fig. 6d) or HMOX1 (Fig. 6c). Overall, these data indicate that the absence of H-ferritin in macrophages leads to an increase in iron export through ferroportin.

**Table 3:** Alterations in gene expression ^a^in *Fth1*^+/+^ and *Fth1*^−/−^ BMDM upon FAC or hemin treatments, at 12 h.

Genes of interest	BMDM genotype	FAC	Hemin
*Fth1*	*Fth1*^+/+^	1.65 ± 0.83	2.94 ± 1.26
	*Fth1*^−/−^	0.11 ± 0.05*	0.10 ± 0.03*
*Ftl*	*Fth1*^+/+^	3.38 ± 2.60	3.57 ± 0.16
	*Fth1*^−/−^	4.61 ± 2.39	5.10 ± 0.27
*Slc40a1*	*Fth1*^+/+^	3.92 ± 2.30	18.19 ± 0.27
	*Fth1*^−/−^	11.53 ± 0.25	18.65 ± 14.23
*Tfrc*	*Fth1*^+/+^	1.39 ± 0.73	0.31 ± 0.14
	*Fth1*^−/−^	1.0. ± 0.60	0.30 ± 0.32
*Hmox1*	*Fth1*^+/+^	6.09 ± 6.70	20.19 ± 7.54
	*Fth1*^−/−^	10.24 ± 9.00	17.07 ± 9.34
*Sod2*	*Fth1*^+/+^	4.05 ± 2.45	4.65 ± 1.47
	*Fth1*^−/−^	4.40 ± 2.08	5.06 ± 2.75
*Cat*	*Fth1*^+/+^	7.36 ± 5.31	11.57 ± 1.87
	*Fth1*^−/−^	14.22 ± 0.42	17.85 ± 3.69
*Trxr*	*Fth1*^+/+^	6.34 ± 5.58	16.33 ± 8.17
	*Fth1*^−/−^	6.16 ± 3.24	11.50 ± 1.98
*Gclc*	*Fth1*^+/+^	1.68 ± 0.71	12.11 ± 7.23
	*Fth1*^−/−^	2.48 ± 0.18	3.64 ± 1.66

^a^The values are calculated as the fold change relative to the *Fth1*^+/+^ non-treated cells, and represent the mean ± SD of three independent cultures per condition; two-way ANOVA *p < 0.05.

### FTH1 protects macrophages against iron-induced oxidative stress

**Figure 7:**
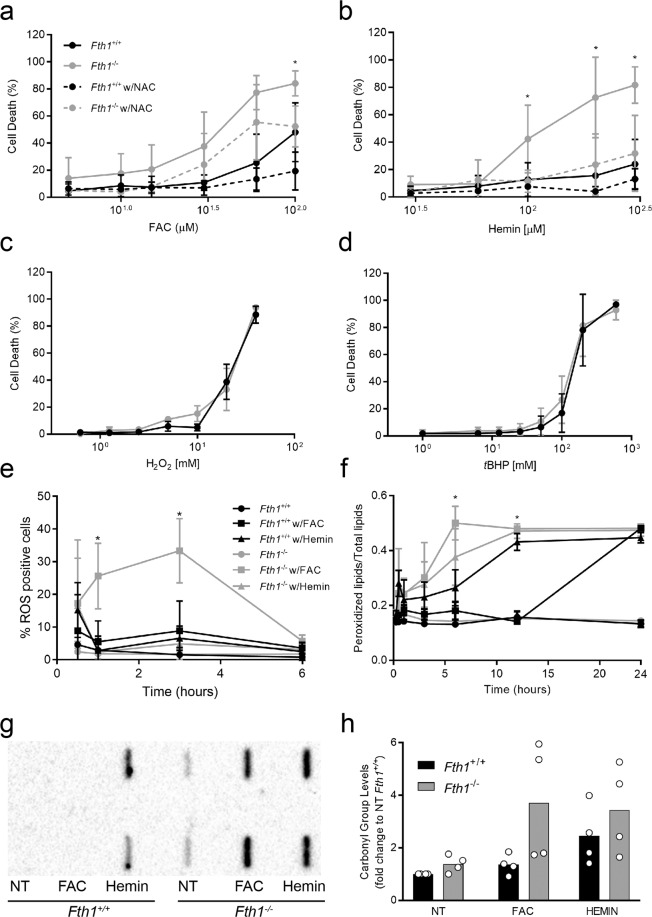
The absence of H-ferritin is associated with higher levels of iron-induced oxidative stress. (**a**,**b**) *Fth1*^+/+^ (black) and *Fth1*^−/−^ (grey) macrophages were treated with increasing concentrations of (**a**) FAC or (**b**) Hemin, in the absence (solid lines) or presence (dashed lines) of NAC, for 3 days. Cell death was measured through SYTOX^TM^ Green incorporation into dead cells. The results represent the mean ± SD of four to seven independent cultures per condition (two-way ANOVA with Tukey multiple comparisons test; *p < 0.05). (**c**,**d**) *Fth1*^+/+^ (black) and *Fth1*^−/−^ (grey) BMDM were treated with increasing concentrations of (**c**) hydrogen peroxide or (**d**) *t*BHP, for 15 minutes. Cell death was analyzed through SYTOX Green incorporation into dead cells. The results represent the mean ± SD of three independent cultures per condition. (**e**,**f**) *Fth1*^+/+^ (black) and *Fth1*^−/−^ (grey) BMDM were kept untreated (circles) or treated with 10 µM FAC (square) or 100 µM hemin (triangle). (**e**) ROS production was analyzed through oxidation of DCFDA dye. The results represent the mean ± SD of three independent cultures per condition and are expressed as the percentage of macrophages marked with the fluorescent-oxidized dye. (**f**) Lipid peroxidation was assessed through BODIPY assay. The results represent the mean ± SD of three independent cultures per condition and are expressed as the ratio between peroxidized lipids (intracellular fluorescence intensity at 510 nm) relative to the total lipids (intracellular fluorescence intensity at 510 nm plus at 590 nm); (two-way ANOVA with Tukey multiple comparisons test; *p < 0.05). (**g**,**h**) *Fth1*^+/+^ (black) and *Fth1*^−/−^ (grey) BMDM were treated with 10 µM FAC or 100 µM hemin for 24 hours. (**g**) Detection of carbonylated proteins by slot blot. The image corresponds to one representative experiment with BMDM obtained from 1 animal per genotype. Cell extracts were blotted in duplicate. The whole membrane is shown. (**h**) Densitometry analysis with ImageLab^TM^ software of carbonylated proteins. The bars represent the mean + SD from 4 independent cultures, of the relative density of the bands obtained from *Fth1*^+/+^ (black) or *Fth1* ^−/−^ (grey) BMDM in each condition, as compared to non-treated *Fth1*^+/+^ cells.

Knowing that *Fth1*-deficient macrophages are more prone to iron-induced death despite lower intracellular iron levels, we investigated whether their increased cell death was caused by oxidative stress. For that, macrophages were incubated with iron together with the antioxidant N-acetyl-cysteine (NAC) and cell death was quantified by incorporation of SYTOX Green. The toxic effect of exogenous iron was partially prevented by the presence of NAC (Fig. 7a,b), indicating that it is, at least partially, mediated by reactive oxygen species. To evaluate whether *Fth1*^−/−^ macrophages had an intrinsically higher susceptibility to oxidative stress, we treated them with either hydrogen peroxide or *tert*-butyl-hydrogen peroxide (*t*BHP). Cell death increased with both treatments in a dose-dependent manner, but no differences were observed between *Fth1*^+/+^ and *Fth1*^−/−^ macrophages (Fig. 7c,d). Altogether, these results (Fig. 7a–d) indicate that both genotypes are able to withstand similar levels of oxidative stress. However, when faced with equal concentrations of iron, the oxidative damage in *Fth1*^−/−^ cells appears to be higher than in *Fth1*^+/+^ cells. To confirm that, we measured the levels of ROS, lipid peroxidation and protein carbonylation. FAC induced a clearly higher level of ROS formation on *Fth1*^−/−^ cells compared to *Fth1*^+/+^ cells, especially 1 and 3 hours after treatment (Fig. 7e). *Fth1*^−/−^ macrophages also had significantly higher levels of lipid peroxidation at 6 and 12 hours after treatment with FAC (Fig. 7f). In the same line, protein carbonylation was higher in iron-treated macrophages of the *Fth1*^−/−^ than *Fth1*^+/+^ genotype (Fig. 7g,h). Interestingly, basal levels of protein carbonylation tended to be higher in non-treated *Fth1* deficient cells, although the difference relative to wild-type was not statistically significant.

In order to understand whether *Fth1*^−/−^ macrophages had more oxidative stress due to some defect in the induction of cell-protective mechanisms, we measured the expression of several genes involved in these mechanisms. *Fth1*^−/−^ macrophages upregulated their expression of *Hmox1*, superoxide dismutase (*Sod2*), catalase (*Cat*), thioredoxin reductase (*Trxr*) and glutamate-cysteine ligase catalytic subunit (*Gclc*) similarly to *Fth1*^+/+^ cells (Table 3).

Overall, the data obtained in this work, clearly indicates that *Fth1* has an essential, non-redundant role in cell protection against iron-induced toxicity.

## Discussion

H-ferritin is crucial for mouse development and survival, with the total knockout being embryonically lethal^9^. In this work, we show that H-ferritin is not necessary for *in vitro* differentiation of murine BMDM nor has a significant impact in these macrophages’ basal state, but it influences macrophage response to immune activation or iron treatments. In particular, H-ferritin-deficient BMDM produce less nitric oxide in response to IFNG + LPS treatment and are more prone to oxidative stress and cell death induced by exogenously added iron.

H-ferritin-deficient BMDM were indistinguishable from wild-type BMDM regarding the kinetics of differentiation, morphology, viability, and the expression of several iron- and activation-related genes. In particular, no significant compensatory increase in L-ferritin expression was found in H-ferritin-deficient macrophages. This is in contrast with the results obtained by Bolisetty *et al*. using the same type of cells^19^. The explanation could be related to differences in culture conditions. Of note, in previous studies, it was observed that transient silencing of *Fth1* expression resulted in upregulation of *Ftl*^20,21^, but stable H-ferritin-deficient HeLa cell clones had normal levels of L-ferritin^21^. Together with the results presented here, this indicates that when cells undergo a long-term adaption to the lack of H-ferritin the expression of L-ferritin is not increased. This adaptation may include a decrease in intracellular iron accumulation, as indicated by our results.

H-ferritin is known to be involved in macrophage activation. When we treated BMDM with IFNG and LPS, the expression of *Fth1* doubled, in agreement with previous results obtained with several TLR agonists^14,22^. An increase in expression of *Fth1* upon IFNG + LPS treatment is apparent in *Fth1*^−/−^ cells (Supplementary Table 2), which is probably related to a minor leakage of *Fth1* expression due to Cre failure, but this remained at residual levels compared to wild-type cells (Fig. 2a). In contrast, *Ftl* expression was not significantly altered by IFNG + LPS treatment, irrespective of *Fth1* expression. In agreement with previous reports^22,23^, the ferroportin-coding gene *Slc40a1* was down-regulated by IFNG + LPS treatment in *wild-type* macrophages. However, in *Fth1*^−/−^ cells, a significant up-regulation of this gene was observed at 24 hours post-treatment, indicating that the expression of this important iron-export gene is regulated by cellular H-ferritin levels. In agreement to what has been described previously by Ludwiczek *et al*. in THP-1 monocytes^24^, wild-type BMDM had a significantly higher expression of the *Tfrc* gene (coding for the transferrin receptor 1, involved in cellular iron uptake) from 24 hours onward after IFNG + LPS treatment. In contrast, in *Fth1*^−/−^ macrophages this gene was down-regulated by treatment at all the time-points analysed. Together, the differential regulation of both ferroportin and transferrin receptor genes in *Fth1*^−/−^ macrophages emphasize the inability of these cells to store iron. Additionally, *Fth1*^−/−^ BMDM had a significantly higher expression of *Hmox1* 24 hours post-treatment, in comparison to *Fth1*^+/+^ macrophages. *Hmox1* gene expression is known to increase in response to ROS generating agents and to be up-regulated in an inflammatory environment, such as mycobacterial infections^25,26^. This increase in *Hmox1* expression indicates that, besides an impairment on iron-retaining capacity, *Fth1*^−/−^ cells exhibit a higher degree of oxidative stress in response to IFNG + LPS treatment. Intriguingly, this increased expression of *Hmox1* occurred concomitantly with a decrease in *Nos2a* expression and nitrite release (Figs. 2 and 3), which is in contrast with a previous study that suggested that *Hmox1* upregulation upon IFNG + LPS treatment was due to increased *Nos2a* expression and nitrite production^25^. Conversely, our data suggest that overall ROS level, rather than NOS2 activity, underlies *Hmox1* induction. Of note, although we did not thoroughly studied the level of ROS in *Fth1*^−/−^ versus *Fth1*^+/+^ BMDM after IFNG + LPS treatment, there was a tendency to have higher levels of carbonylated proteins in *Fth1*^−/−^ cells under basal conditions (Fig. 7g). The clear dampening of *Nos2a* gene induction in the absence of H-ferritin and in a situation of low intracellular iron level, was also an intriguing observation *per se* and corroborates a previous observation by Bolisetty *et al*.^19^. Several previous studies showed that treating macrophages with excess iron decreased *Nos2a* expression and nitrite production upon treatment with IFNG and/or LPS^27–29^. We now postulate that these effects were due to increased intracellular oxidative stress rather than to a direct effect of iron. Different signalling pathways have been implicated in the relationship between iron and *Nos2a* expression, including signal transducer and activator of transcription 1 (STAT1)^27^ and hypoxia-inducible factor 1-alpha (HIF1A)^30^. The intracellular iron levels, labile iron pool (LIP) and Iron Responsive Proteins (IRP) have also been implicated^31^. The exact mechanisms linking the lack of H-ferritin to the modulation of *Nos2a* expression are beyond the scope of this work but will be the subject of future studies.

H-ferritin is an essential player in iron metabolism since it has iron storage and detoxifier activities. To understand the importance of H-ferritin in this scenario, we treated macrophages with exogenous iron in the forms of FAC and hemin. In agreement with previous reports^25,28^, both forms of iron caused a dose-dependent reduction in macrophages’ viability (and an increase in cell death). However, there was a clearly higher susceptibility to exogenous iron in the absence of *Fth1*. We reasoned that this occurred because without *Fth1*, cells are unable to oxidize Fe^2+^, and this will accumulate in a labile reactive pool. Previous studies have indicated that in the absence of H-ferritin cells have an increased LIP^20,32,33^. In order to have an insight on the alterations in intracellular iron distribution caused by the lack of H-ferritin in our model, we used electron microscopy coupled with EDS. To our surprise, *Fth1*^−/−^ BMDM had significantly less intracellular iron than normal BMDM, making it technically impossible to quantify iron in different intracellular compartments. This decrease in intracellular iron may be caused by an inability to store iron in a normal ferritin nanocage in the absence of FTH1^34^, as well as a tendency to have increased levels of ferroportin, the only known protein exporter. In accordance with this finding, studies on the tissue iron distribution in *Fth*^*lox/lox*^; MxCre mice revealed that the lack of FTH1 results in decreased iron storage in the liver and spleen^17^.

Despite the decrease in the amount of intracellular iron, *Fth1*-deficient BMDM had increased levels of oxidative stress when exposed to exogenous iron, as evaluated by the levels of ROS, lipid peroxidation and protein carbonylation. Our data indicate that iron-induced oxidative stress is the main cause of increased cell death in the *Fth1*^−/−^ macrophages upon iron treatment since in the presence of NAC, *Fth1*^−/−^ cells were as protected from cell death as *Fth1* sufficient cells. As mentioned before, this effect is probably due to the vulnerability of *Fth1*^−/−^ cells to Fe^2+^ as they lack the ferroxidase activity conferred by H-ferritin. Importantly, the lack of H-ferritin did not increase the susceptibility of BMDM to different peroxide sources, indicating that H-ferritin is not directly involved in peroxide detoxification, but it is involved in decreasing peroxide formation from iron. Furthermore, the data show that macrophages do not have an alternative mechanism to deal with iron-mediated oxidative stress, even though they can upregulate the transcription of several genes involved in the response to oxidative stress. Overall, the alterations seen in *Fth1*^−/−^ macrophages are consistent with the recently described type of programmed cell death, “ferroptosis”^35–38^, although the exact mechanisms of cell death were not thoroughly characterized here.

In conclusion, this work shows that although H-ferritin is not essential for the *in vitro* differentiation of bone marrow-derived macrophages, it modulates macrophage activation by immune and microbial stimuli and, more importantly, it has a non-redundant role in cell protection against iron toxicity.

## Materials and Methods

### Chemicals

All chemicals were obtained from Sigma-Aldrich (St. Louis, MO, USA), unless otherwise specified.

### Macrophage cultures

Bone marrow-derived macrophages (BMDM) were obtained as described previously^39^ from the bone marrow of *Fth1*^+/+^ or *Fth1*^−/−^ mice.

Mice referred to as *Fth1*^−/−^ mice are conditional *Fth1* deficient (*Fth1*^Fl/Fl^; *Lyz2*^*cre/+*^) mice, obtained by crossing *Fth1*^Fl/Fl^ mice^17^ with *Lyz2*^*cre/+*^ mice. An initial breeding pair was kindly provided by Prof. Lukas Kuhn (Swiss Institute for Experimental Cancer Research, Lausanne, Switzerland). In these mice, Cre recombinase deletes the *Fth1* gene in cells expressing *Lyz2* (cells of the myeloid lineage). *Fth1*^Fl/Fl^; Lyz2^+/+^ Cre-negative littermate mice were used as controls and will be referred to as *Fth1*^+/+^ mice.

All experimental animal procedures described in this work were approved by the Local Animal Ethics Committee of IBMC/i3S and licensed by the Portuguese Authority “General Directory of Agriculture and Veterinary” (DGAV), on July 6th, 2016 with reference 0421/000/000/2016. All animals were handled in strict accordance with good animal practice as defined by national authorities (DGAV, Decreto-Lei 113/2013, august 7th) and European Directive 2010/63/EU.

Cell cultures were visualized and imaged every day during the experiment in an inverted optical microscope (Leica DMI6000 Timelapse).

For macrophage activation experiments, recombinant mouse IFNG (Gibco, MD, USA) was added at 16 ng/ml, at the 7th day of culture. For kinetic studies, IFNG was added two additional times thereafter with 24 h intervals. Lipopolysaccharide (LPS) was given once at 10 ng/ml.

For iron manipulation experiments, ferric ammonium citrate (FAC) or hemin (Frontier Scientific Inc, Logan, UT, USA) were added to the cells in complete medium on the 7th day of culture, at the concentrations indicated in the results section. FAC was dissolved in PBS, and hemin was prepared as previously described^25^.

### Evaluation of macrophage differentiation by flow cytometry

Cells were collected with cold 5 mM EDTA/PBS on the 7^th^ and 10^th^ day of culture, incubated with anti-Fc receptor blocking antibody (BioLegend CD16/32) and then stained with BV510-conjugated anti-mouse CD11b and APC-Cy7-conjugated anti-mouse F4/80 antibodies (1:200 and 1:100, respectively; Biolegend, San Diego, CA, USA) in FACS buffer. Unstained cells were used as negative controls to adjust the machine settings before acquisition. Samples were analyzed using the BD FACSCanto^TM^ II Bioanalyser (BD Biosciences, San Jose, CA, USA) and with FlowJo software (FlowJo, LLC, Ashland, OR, USA). Cells were initially selected based on the size (forward scatter, FSC) and granularity (side scatter, SSC). Double positive cells for F4/80 and CD11b were considered mature macrophages.

### Protein expression quantification by western blot

All the procedures were performed at 4 °C. Cells were harvested with cold 5 mM ethylenediaminetetraacetic (EDTA)/PBS and resuspended in collecting buffer (Ripa buffer: 150 mM NaCl, 1.0% NP-40, 0.5% sodium deoxycholate, 0.1% SDS, 50 mM Tris, pH 8.0), supplemented with dithiothreitol (DTT), phenylmethylsulfonyl fluoride (PMSF) and a protease inhibitor cocktail. The protein extract was then stored at −80 °C, prior to sample quantification with DC^TM^ protein assay (Bio-Rad, Hercules, CA, USA) and preparation for western and slot blot.

Equivalent amounts of protein prepared in Laemmli buffer (Bio-Rad) were separated by electrophoresis in 10% or 12% SDS polyacrylamide gels (SDS-PAGE) and electrophoretically transferred into an activated polyvinylidene difluoride (PVDF) membrane (Fig. 1) (GE Healthcare Life Sciences, USA) or nitrocellulose membrane (Fig. 6), (GE Healthcare Life Sciences, USA). One gel and membrane were prepared for each of the proteins of interest: FTH1 and FTL. From each membrane, the region expected to contain the protein of interest and the region expected to contain the loading control beta-actin (ACTB), based on molecular weight (precision plus protein^TM^ standards, Bio-Rad) were cut. Each piece of the membrane was blocked with 5% bovine serum albumin (BSA) in Tris-buffered saline with Tween-20 (TBS-T) (NaCl 150 mM, Tris 50 mM, Tween-20 0.1%) followed by overnight incubation with the primary antibody (anti-FTH1, 1:500, catalog #4394, Cell Signaling Technology, Danvers, MA, USA; anti-FTL, 1:1000, catalog #ab69090, Abcam Cambridge, UK; anti-ACTB, 1:5000, catalog #ab8227, Abcam, Cambridge, UK, anti-SLC40A1, 1:500, catalog # NBP1-21502, Novus Biologicals, CO, US, anti-HMOX1, 1:400, catalog #10701-1-AP, Proteintech, Deansgate, UK) prepared in 1% BSA/TBS-T. The specificity of each antibody was confirmed in preliminary assays performed on the entire membrane. The membranes were then incubated with the secondary antibody-horseradish peroxidase (HRP) (anti-rabbit, 1:10000, catalog# 111-035-144, Jakson ImmunoResearch, Cambridgeshire, UK) in 1% BSA/TBS-T. The specificity of each antibody was confirmed in preliminary assays performed on the entire membrane. The membranes were then incubated with the secondary antibody-horseradish peroxidase (HRP) (anti-rabbit, 1:10000, The Binding Site, Birmingham, UK) in 1% BSA/TBS-T. Membranes were imaged in ChemiDoc (Bio-Rad), in the presence of HRP substrate (Luminata^TM^ Milipore, Billerica, MA, USA). Densitometry analysis was performed with ImageLab^TM^ software (Bio-Rad).

### Evaluation of oxidative protein damage by slot blot

Protein samples obtained as described above were derivatized with 2,4-dinitrophenylhydrazine (DNPH) into 2,4-dinitrophenyl (DNP), following the method of Levine and collaborators (Levine, Garland *et al*. 1990). Equivalent protein amounts from the derivatized samples were applied to a PVDF membrane using a slot-blot apparatus (Hybri.Slot 24, Core Life Sciences, Irvine, CA, USA) and then the membranes were incubated overnight at 4 °C with a rabbit anti-DNP antibody (1:5000, Sigma–Aldrich, St. Louis, MO, USA). Samples were marked using a goat anti-rabbit IgG HRP conjugate (1:5000, Sigma-Aldrich, St. Louis, MO, USA). Membranes were imaged using a Bio-Rad FX-Pro-plus (Bio-Rad, Hemel Hempstead, UK) with HRP (Luminata^TM^ Milipore, Billerica, MA, USA) substrate.

### Analysis of gene expression

RNA was obtained from BMDM using the PureLink® RNA Mini Kit, and following the manufacturer’s instructions (Ambion^TM^, Invitrogen, Carlsbad, CA, USA). Total RNA was transcribed into cDNA by using an NZY First-Strand cDNA synthesis kit (NZYTech, Lisbon, Portugal). For the amplification of each gene of interest, a corresponding specific pair of primers (STAB Vida, Lisbon, Portugal) was used (Table 4). All reactions were performed in a 20 μL total volume with iTaq SYBR green PCR master mix (Bio-Rad, Hercules, CA, USA).

**Table 4:** Primers sequences.

Gene and nomenclature	Primer	Sequence
Hypoxanthine Phosphoribosyltransferase (*Hprt*)	*Hprt* forward	5′-GGTGGAGATGATCTCTCAAC-3′
	*Hprt* reverse	5′-TCATTATAGTCAAGGGCATATCC-3′
H-ferritin (*Fth1*)	*Fth1* forward	5′-GCTGAATGCAATGGAGTGTGCA-3′
	*Fth1* reverse	5′-GGCACCCATCTTGCGTAAGTTG-3′
L-ferritin (*Ftl*)	*Ftl* forward	5′-ACCTACCTCTCTCTGGGCTT-3′
	*Ftl* reverse	5′-TGGCTTCTGCACATCCTGGA-3′
Ferroportin (*Slc40a1*)	*Slc40a1* forward	5′-TTGGTGACTGGGTGGATAAGAATGC-3′
	*Slc40a1* reverse	5′-CGCAGAGGATGACGGACACATTC-3′
Heme oxygenase 1 (*Hmox1*)	*Hmox1* forward	5′-GCCACCAAGGAGGTACACAT-3′
	*Hmox1* reverse	5′-GCTTGTTGCCCTCTATCTCC-3′
Transferrin receptor (*Tfrc*)	*Tfrc* forward	5′-GCAGCATTGGTCAAAACATGG-3′
	*Tfrc* reverse	5′-GCTTTGGGCATTTGCAACCC-3′
Inducible nitric oxyde synthase (*Nos2a*)	*Nos2a* forward	5′-ACATCGACCCGTCCACAGTAT-3′
	*Nos2a* reverse	5′-CAGAGGGGTAGGCTTGTCTC-3′
Arginase 1 (*Arg1*)	*Arg1* forward	5′-CTCCAAGCCAAAGTCCTTAGAG-3′
	*Arg1* reverse	5′-CGAGCTGTCATTAGGGACATCA-3′
Tumor necrosis factor (*Tnf*)	*Tnf* forward	5′-CCGTCAGCCGATTTGCTATCT-3′
	*Tnf* reverse	5′-CGGACTCCGCAAAGTCTAAG-3′
Superoxide Dismutase (*Sod2*)	*Sod2* forward	5′-GTCGCTTACAGATTGCTGCCTGCT-3′
	*Sod2* reverse	5′-GTGCTCCCACACGTCAATCCC-3′
Catalase (*Cat*)	*Cat* forward	5′-TCACCGGCACATGAATGGCTATGGA-3′
	*Cat* reverse	5′-TGCCCTGGTCGGTCTTGTAATGGA-3′
Thioredoxin reductase (*Trxr*)	*Trxr* forward	5′-CAGGGTGACTGCTCAATCCACAAAC-3′
	*Trxr* reverse	5′-CTCTTCCTACCGCCAGCAACACTG-3′
Glutamate-cysteine ligase catalytic subunit (*Gclc*)	*Gclc* forward	5′-TCACCGGCACATGAATGGCTATGGA-3′
	*Gclc* reverse	5′-TGCCCTGGTCGGTCTTGTAATGGA-3′

Baseline thresholds were obtained by using the Bio-Rad iQ5 program and the threshold cycles (CTs) were calculated by the 2^CT^ method, where CT values for the genes of interest were normalized to the level of the hypoxanthine-guanine phosphoribosyl transferase housekeeping gene (*Hprt1*). Data are shown as n-fold differences relative to values for non-treated samples or for wild-type genotype, calculated with the 2^−ΔΔCT^ method.

### Evaluation of cell viability

Cell metabolic activity was measured by resazurin reduction. Briefly, 10% (v/v) of resazurin (0.3 mg/ml) was added to the cells and incubated for 20 h. The fluorescence of resorufin, resulting from the reduction of resazurin by metabolically active cells, was measured at λ_ex_ = 530 nm and λ_em_ = 590 nm in Synergy^TM^ Mx (BioTek, Winooski, VT, USA).

For the evaluation of membrane permeability, cells were incubated with a mixture of a cell-permeable nuclear dye Hoechst 33342 (1:12000, Invitrogen, Eugene, OR, USA), and a membrane-impermeable dye SYTOX^TM^ Green (1:45000, Thermo Fisher Scientific, Waltham, MA, USA), for 20 minutes at 37 °C and 7% CO_2_. Cells were visualized in a 37 °C, CO_2_ atmosphere with a 20× Nikon objective in a high-throughput automated fluorescence wide-field microscope (IN Cell Analyzer 2000, GE Healthcare, Little Chalfont, UK). The acquired images were then analyzed with Developer Toolbox 1.9.2 (GE Healthcare).

### Quantification of nitrite production

After 72 h of incubation with the different stimuli, the macrophage culture supernatant was recovered, and incubated with Griess reagent (1% sulfanidamine, 0.1% naphthylenediamine dihydrochloride (NEDADHC), 1.72% phosphoric acid 85%, in water) in 1:1 proportion, for 10 minutes. A standard curve was made using sodium nitrite (NaNO_2_). After incubation, the absorbance was read at 550 nm on a µQuant^TM^ Microplate Spectrophotometer (BioTek Instruments, Winooski, VT, USA).

### Quantification of TNFa by ELISA

Tumor Necrosis Factor alpha (TNFa) was quantified in macrophage culture supernatant 72 h after treatments, using ELISA Ready-SET-Go (Thermo Fisher Scientific, Waltham, MA, USA) according to the manufacturer’s instructions.

### Evaluation of oxidative stress

ROS production was evaluated using 2′,7′-dichlorofluorescein diacetate (DCFDA), as described previously^40^. The oxidation of the dye by ROS generates a highly fluorescent compound, 2,7-dichlorofluorescein (DCF) that can be detected at λ_ex_ = 504 nm and λ_em_ = 529 nm. Briefly, cells were incubated with DCFDA (5 µM) for 30 minutes, washed with warm PBS/5% FBS, and then imaged in a 37 °C, CO_2_ atmosphere with a 20× Nikon objective in a high-throughput automated fluorescence wide-field microscope (IN Cell Analyzer 2000, GE Healthcare, Little Chalfont, UK). The acquired images were then analyzed with Developer Toolbox 1.9.2 (GE Healthcare).

Lipid peroxidation was measured with BODIPY®, following the manufacturer’s instructions (Thermo Fisher Scientific). Upon oxidation by lipid hydroperoxides, BODIPY® changes its maximum fluorescence emission wavelength from 590 nm to 510 nm. Briefly, cells were incubated with BODIPY® (10 µM) for 30 minutes, washed with PBS, and then visualized in a 37 °C, CO_2_ atmosphere with a 20× Nikon objective in a high-throughput automated fluorescence wide-field microscope (IN Cell Analyzer 2000, GE Healthcare). The acquired images were analyzed with CellProfiler^TM^.

### Iron quantification

Iron levels were measured through transmission electron microscopy (TEM) – energy-dispersive X-ray spectroscopy (EDS) and through atomic absorption spectrometry. Sample processing for TEM was performed as described previously^41^. For EDS analysis, the sections were mounted on nickel grids and a beryllium holder (EM-21150, Jeol Ltd.) was used. An X-Max 80 mm^2^ (Oxford Instruments, Bucks, England) operated at 120 kV was coupled to the microscope, at the HEMS/i3S of the Universidade do Porto, Portugal.

For iron quantification by atomic absorption spectrometry, cells were collected 24 hours after iron treatments. Pellets were dried at 65 °C overnight and digested with 69% nitric acid. The measurements were performed on an AAnalyst 200 (PerkinElmer, Waltham, MA) flame atomic absorption spectrometer, with a hollow cathode lamp (SCP Science, Quebec, Canada) as the radiation source. The absorbance was read at 248.33 nm in an air (10 L/min) - acetylene (2.5 L/min) flame. Data were normalized using the phosphorous content in each sample.

Phosphorus (^31^P) determination was performed through inductively coupled plasma-mass spectrometry (ICP-MS) using an iCAP™ Q (Thermo Fisher Scientific, Bremen, Germany) instrument, equipped with a Meinhard^®^ (Golden, Colorado) TQ^+^ high sensitivity nebulizer, a baffled cyclonic spray chamber (Peltier-cooled), a standard quartz torch, and a two-cone design (nickel sample and skimmer cones). High purity (99.9997%) argon (Gasin, Matosinhos, Portugal) was used as a nebulizer and as a plasma gas source. The ICP-MS instrument operational parameters were as follows: RF power (1550 W); plasma gas flow (14 L/min); auxiliary gas flow (0.8 L/min); nebulizer flow rate (1.02 L/min). The equipment control and data acquisition were made through the Qtegra™ software (Thermo Fisher Scientific). The elemental isotope ^45^Sc was used as an internal standard.

### Statistical analysis

Data analysis was performed using GraphPad Prism 7.0 program (GraphPad Software, Inc., La Jolla, CA, USA), and data were expressed as means plus standard deviations of the number of samples indicated in the legend of each figure. Multiple comparisons were performed by using two-way analysis of variance (ANOVA) followed by a Tukey multiple-comparison post hoc test. Comparisons between two groups were performed by using Student’s t test. In both cases, differences were considered significant when the P value was <0.05.

## Supplementary information

Supplementary Information.
