# Supplementary material for: H-Ferritin is essential for macrophages’ capacity to store or detoxify exogenously added iron
Source: Sci Rep. 2020 Feb 20;10:3061. doi: 10.1038/s41598-020-59898-0 (PMC7033252; doi:10.1038/s41598-020-59898-0)
Supplement: Supplementary file 1 — Supplementary Information. [file 41598_2020_59898_MOESM1_ESM.docx]

Supplementary Information

**H-Ferritin is essential for macrophages’ capacity to store or detoxify exogenously added iron**

**Gonçalo Mesquita ^1,2†^, Tânia Silva^1,2†^, Ana C. Gomes^1,2,3^, Pedro F. Oliveira^1,4,5^, Marco G. Alves^4^, Rui Fernandes^1,2^, Agostinho A. Almeida^6^, Ana C. Moreira ^1,2,3^*^¶^ and Maria Salomé Gomes ^1,2,3,^*^¶^**

^1^ i3S – Instituto de Investigação e Inovação em Saúde, Universidade do Porto, Portugal;

^2^ IBMC – Instituto de Biologia Molecular e Celular, Universidade do Porto, Portugal;

^3^ Departamento de Biologia Molecular, ICBAS – Instituto de Ciências Biomédicas Abel Salazar, Universidade do Porto, Portugal;

^4^ Department of Microscopy, Laboratory of Cell Biology and Unit for Multidisciplinary Research in Biomedicine (UMIB), ICBAS, Universidade do Porto, Portugal;

^5^ Department of Genetics, Faculty of Medicine (FMUP), Universidade do Porto, Portugal;

^6^ LAQV / REQUIMTE, Departamento de Ciências Químicas, Faculdade de Farmácia, Universidade do Porto, Portugal;

† These authors contributed equally to this work

^¶^ These authors contributed equally to this work

***** Correspondence: ana.s.moreira@ibmc.up.pt (ACM); sgomes@ibmc.up.pt (MSG)


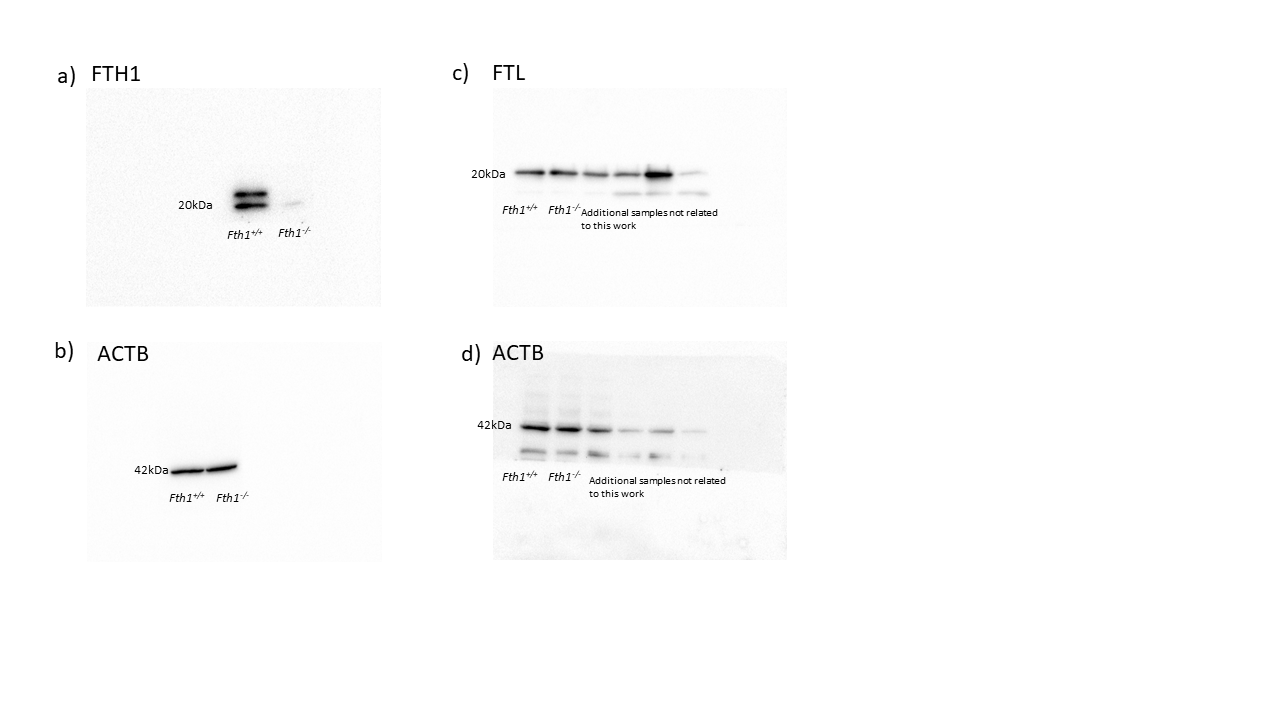


**Supplementary figure 1: Raw images from western blot membranes used for ferritin quantification in cell extracts.**

BMDM cell extracts were separated by SDS-PAGE and electrophoretically transferred into an activated polyvinylidene difluoride (PVDF) membrane. One gel and membrane were prepared for each of the proteins of interest: FTH1 (a) and c)) and FTL (b) and d)). From each membrane, the region expected to contain the protein of interest and the region expected to contain the loading control beta-actin (ACTB), based on molecular weight, were cut. Each piece of the membrane was blocked and incubated with the primary antibody: FTH1 (a); FTL (c) or ACTB (b,d). The specificity of each antibody was confirmed in preliminary assays performed on the entire membrane. The membranes were then incubated with the secondary antibody-horseradish peroxidase (HRP). Membranes were imaged in ChemiDoc, in the presence of HRP substrate.

In order to compose the manuscript main Figure 1 (**a**), we cropped the initial images and zoomed in. No alterations in the initial brightness or contrast were made.

**
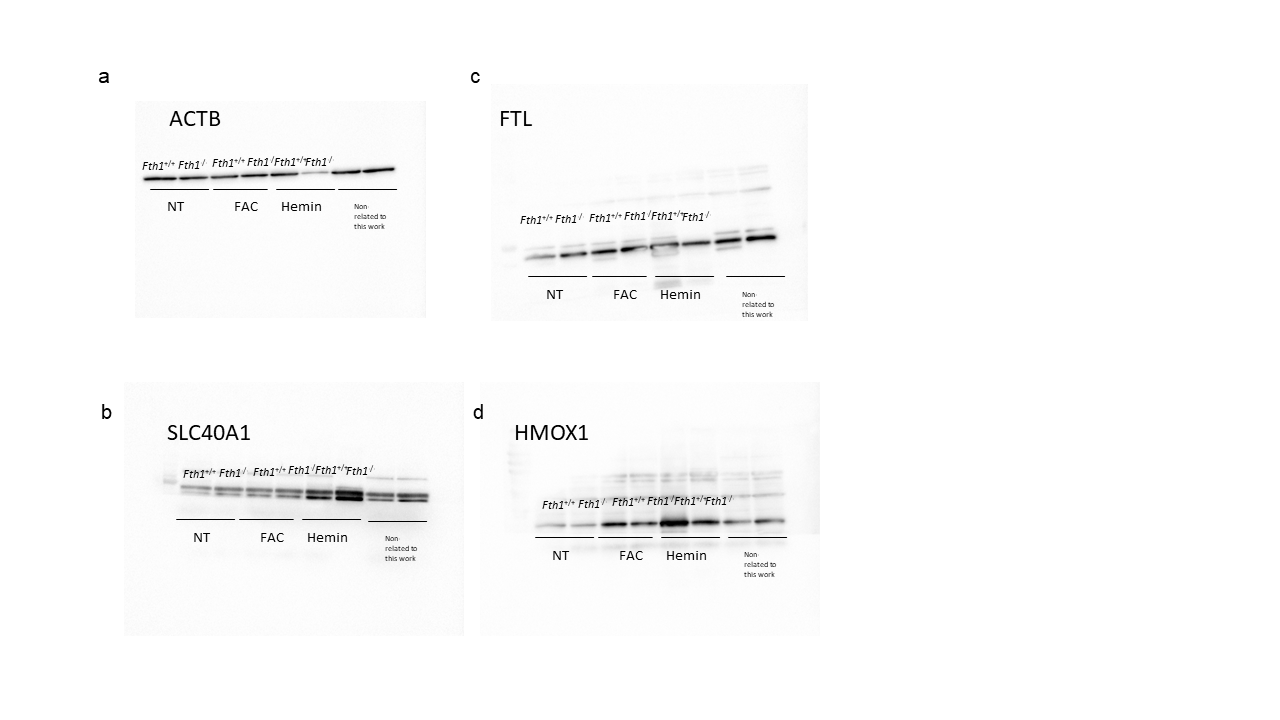
**

**Supplementary figure 2: Raw images from western blot membranes used for SLC40A1, HMOX1 and FTL quantification in cell extracts upon treatment with FAC or hemin.**

BMDM cell extracts were separated by SDS-PAGE and electrophoretically transferred onto a nitrocellulose membrane. Each piece of the membrane was blocked and incubated with the primary antibody: ACTB (a); SLC40A1 (b) FTL (c) or HMOX1 (b,d). The membranes were then incubated with the secondary antibody-horseradish peroxidase (HRP). Membranes were imaged in ChemiDoc, in the presence of HRP substrate.

In order to compose the manuscript main Figure 6, we cropped the initial images and zoomed in. No alterations in the initial brightness or contrast were made.

**
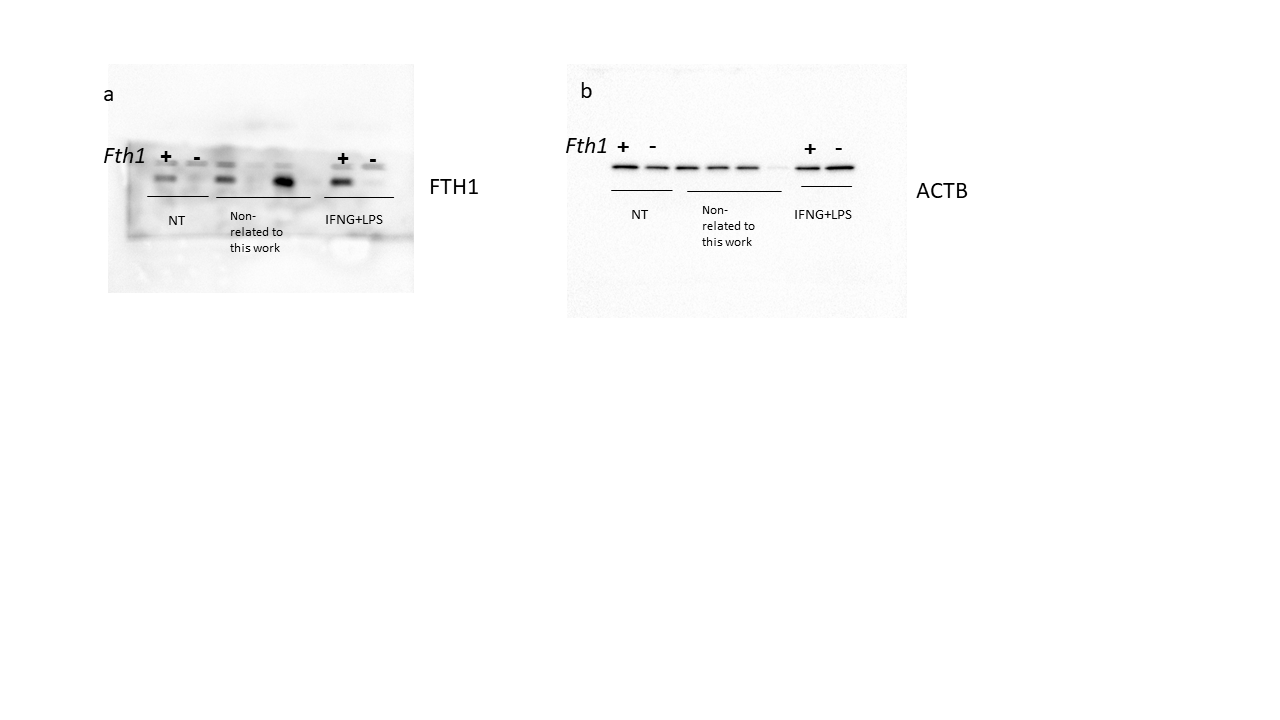
Supplementary figure 3: Raw images from western blot membranes used for FTH1 expression**

BMDM cell extracts were separated by SDS-PAGE and electrophoretically transferred onto a PVDF membrane. Each piece of the membrane was blocked and incubated with the primary antibody: FTH1 (a) and ACTB (b). The membranes were then incubated with the secondary antibody-horseradish peroxidase (HRP). Membranes were imaged in ChemiDoc, in the presence of HRP substrate.

**Supplementary Table 1.** Alterations in gene expression ^a^ upon IFNG + LPS treatment, at 12, 24 and 72 h.

| **Gene of interest** | **BMDM genotype** | **12 hours** | **24 hours** | **72 hours** |
| --- | --- | --- | --- | --- |
| *Fth1* | *Fth1^+/+^* | 4.07 ± 2.16 | 11.09 ± 4.62 | 2.23 ± 1.90 |
|  | *Fth1^-/-^* | 4.47 ± 2.05 | 17.16 ± 5.84 | 2.21 ± 1.47 |
| *Ftl* | *Fth1^+/+^* | 0.68 ± 0.11 | 2.38 ± 0.81 | 0.80 ± 0.75 |
|  | *Fth1^-/-^* | 0.61 ± 0.02 | 5.09 ± 2.28 | 1.33 ± 1.26 |
| *Slc40a1* | *Fth1^+/+^* | 0.49 ± 0.08 | 0.93 ± 0.12 | 0.17 ± 0.09 |
|  | *Fth1^-/-^* | 0.54 ± 0.29 | 2.27 ± 0.52 | 0.16 ± 0.11 |
| *Hmox1* | *Fth1^+/+^* | 2.96 ± 0.18 | 17.99 ± 5.89 | 6.19 ± 3.08 |
|  | *Fth1^-/-^* | 1.51 ± 0.28 | 24.51 ± 4.73 | 6.62 ± 2.95 |
| *Tfrc* | *Fth1^+/+^* | 0.45 ± 0.20 | 0.89 ± 0.50 | 2.98 ± 0.48 |
|  | *Fth1^-/-^* | 0.35 ± 0.19 | 0.68 ± 0.07 | 0.60 ± 0.17 |
| *Nos2a* | *Fth1^+/+^* | 1573.49 ± 157.06 | 4128.21 ± 4324.24 | 41810.56 ± 27572.08* |
|  | *Fth1^-/-^* | 415.72 ± 151.37 | 1715.56 ± 1527.23 | 3332.87 ± 4562.59 |

^a^ The values were calculated as the fold change relative to each non-treated cells, and represent the mean ± SD of three cultures per condition (two-way ANOVA; *p<0.05).

**Supplementary Table 2.** Alterations in gene expression ^a^ in *Fth1^+/+^* and *Fth1^-/-^* BMDM upon FAC or hemin treatments, at 12 h.

| **Genes of**  **interest** | **BMDM**  **genotype** | **FAC** | **Hemin** |
| --- | --- | --- | --- |
| *Fth1* | *Fth1^+/+^* | 1.65 ± 0.83 | 2.94 ± 1.26 |
|  | *Fth1^-/-^* | 4.06 ± 4.89 | 2.63 ± 1.94 |
| *Ftl* | *Fth1^+/+^* | 3.38 ± 2.60 | 3.57 ± 0.16 |
|  | *Fth1^-/-^* | 2.26 ± 0.41 | 2.03 ± 1.96 |
| *Slc40a1* | *Fth1^+/+^* | 3.92 ± 2.30 | 18.19 ± 0.27 |
|  | *Fth1^-/-^* | 5.36 ± 2.94 | 13.1 ± 12.1 |
| *Trfc* | *Fth1^+/+^* | 1.39 ± 0.73 | 0.31 ± 0.14 |
|  | *Fth1^-/-^* | 1.42 ± 1.44 | 0.49 ± 0.64 |
| *Hmox1* | *Fth1^+/+^* | 6.09 ± 6.70 | 20.19 ± 7.54 |
|  | *Fth1^-/-^* | 8.77 ± 2.62 | 18.4 ± 10.0 |
| *Sod2* | *Fth1^+/+^* | 4.05 ± 2.45 | 4.65 ± 1.47 |
|  | *Fth1^-/-^* | 3.76 ± 1.14 | 4.17 ± 1.43 |
| *Cat* | *Fth1^+/+^* | 7.36 ± 5.31 | 11.57 ± 1.87 |
|  | *Fth1^-/-^* | 4.22 ± 3.65 | 4.80 ± 3.82 |
| *Trxr* | *Fth1^+/+^* | 6.34 ± 5.58 | 16.33 ± 8.17 |
|  | *Fth1^-/-^* | 3.67 ± 1.70 | 18.5 ± 19.0 |
| *Gclc* | *Fth1^+/+^* | 1.68 ± 0.71 | 12.11 ± 7.23 |
|  | *Fth1^-/-^* | 9.1 ± 13.1 | 5.72 ± 6.00 |

^a^ The values are calculated as the fold change relative to the non-treated cells, and represent the mean ± SD of three independent cultures per condition.

**Supplementary Table 3.** Alterations in gene expression ^a^ in *Fth1^+/+^* and *Fth1^-/-^* BMDM upon FAC, hemin, or IFNG+LPS treatments.

| Time-point (h) | BMDM genotype | Treatment | Gene of interest | | | | | | | | | | | | |
| --- | --- | --- | --- | --- | --- | --- | --- | --- | --- | --- | --- | --- | --- | --- | --- |
|  |  |  | ***Hprt*** | ***Fth1*** | ***Ftl*** | ***Slc40a1*** | ***Hmox1*** | ***Tfrc*** | ***Nos2a*** | ***Arg1*** | ***Tnf*** | ***TrxR*** | ***Glcl*** | ***Sod2*** | ***Cat*** |
| 12 | *Fth1^+/+^* | NT | 22,46 ± 1,29 | 14,17 ± 0,79 | 16,33 ± 0,41 | 26,34 ± 0,44 | 20,71 ± 0,48 | 27,49 ± 0,98 | 29,47 ± 1,89 |  |  | 25,43 ± 1,24 | 24,84 ± 0,55 | 22,83 ± 0,87 | 27,31 ± 1,87 |
|  | *Fth1^-/-^* | NT | 21,35 ± 0,49 | 18,28 ± 0,75 | 15,55 ± 0,39 | 25,83 ± 0,26 | 19,02 ± 1,28 | 28,25 ± 1,86 | 27,08 ± 0,88 |  |  | 26,06 ± 1,57 | 24,58 ± 0,47 | 23,18 ± 1,11 | 26,50 ± 1,72 |
|  | *Fth1^+/+^* | IFN+LPS | 22,42 ± 0,60 | 12,24 ± 0,59 | 16,25 ± 1,33 | 26,56 ± 1,56 | 18,23 ± 0,81 | 29,47 ± 2,00 | 18,82 ± 0,33 |  |  |  |  |  |  |
|  | *Fth1^-/-^* | IFN+LPS | 21,84 ± 0,65 | 17,29 ± 0,43 | 16,21 ± 1,63 | 27,32 ± 1,39 | 19,29 ± 1,18 | 30,80 ± 0,45 | 18,94 ± 0,90 |  |  |  |  |  |  |
|  | *Fth1^+/+^* | FAC | 21.38 ± 1,74 | 14,95 ± 1,44 | 16,83 ± 1,90 | 24,98 ± 2,91 | 19,02 ± 1,06 | 28,69 ± 0,58 | 35,06 ± 0,13 |  |  | 25,18 ± 1,54 | 24,77 ± 0,98 | 22,58 ± 1,17 | 26,87 ± 3,22 |
|  | *Fth1^-/-^* | FAC | 21,23 ± 3,74 | 20,46 ± 1,48 | 17,16 ± 0,99 | 24,29 ± 1,71 | 18,53 ± 0,78 | 30,01 ± 1,21 | 36,30 ± 1,81 |  |  | 24,56 ± 1,61 | 25,46 ± 1,49 | 24,25 ± 1,80 | 26,56 ± 3,42 |
|  | *Fth1^+/+^* | Hemin | 21,86 ± 2,38 | 13,28 ± 2,40 | 14,85 ± 2,13 | 23,99 ± 1,89 | 16,56 ± 1,60 | 26,69 ± 0,56 | 34,62 ± 0,12 |  |  | 23,58 ± 1,71 | 23,88 ± 0,51 | 22,02 ± 1,24 | 25,37 ± 3,58 |
|  | *Fth1^-/-^* | Hemin | 21,78 ± 2,55 | 18,80 ± 2,40 | 14,79 ± 1,14 | 22,56 ± 2,23 | 16,76 ± 2,35 | 28,49 ± 2,05 | 34,99 ± 2,08 |  |  | 24,06 ± 2,43 | 23,74 ± 2,71 | 22,72 ± 1,96 | 26,24 ± 4,05 |
| 24 | *Fth1^+/+^* | NT | 20,22 ± 0,14 | 13,09 ± 0,24 | 15,15 ± 0,17 | 24,61 ± 1,17 | 20,66 ± 0,44 | 27,78 ± 0,385 | 29,17 ± 1,06 |  |  |  |  |  |  |
|  | *Fth1^-/-^* | NT | 19,94 ± 0,44 | 19,00 ± 0,19 | 15,11 ± 0,73 | 24,29 ± 1,46 | 19,95 ± 0,13 | 28,85 ± 1,28 | 29,46 ± 0,82 |  |  |  |  |  |  |
|  | *Fth1^+/+^* | IFN+LPS | 20,90 ± 0,60 | 10,41 ± 0,76 | 14,64 ± 1,10 | 25,40 ±1,50 | 17,48 ± 0,67 | 28,87 ± 1,18 | 18,39 ± 1,00 |  |  |  |  |  |  |
|  | *Fth1^-/-^* | IFN+LPS | 21,51 ± 0,41 | 16,54 ± 0,38 | 14,46 ± 1,42 | 24,70 ± 1,10 | 17,07 ± 0,06 | 30,39 ± 1,14 | 19,37 ± 1,22 |  |  |  |  |  |  |
| 72 | *Fth1^+/+^* | NT | 23,12 ± 1.09 | 14,78 ± 0,93 | 16,95 ± 1,49 | 25,52 ± 2,51 | 22,04 ± 1.16 | 29,95 ± 2.16 | 32,95 ± 3.8 | 37,75 ± 1.79 | 25,82 ± 1.22 |  |  |  |  |
|  | *Fth1^-/-^* | NT | 21,21 ± 0,86 | 19,09 ± 1,11 | 15,74 ± 1,12 | 24,01 ± 2,83 | 20,90 ± 1,41 | 28,57 ± 2,44 | 32,63 ± 4,42 | 36,99 ± 1,21 | 24,82 ± 0,27 |  |  |  |  |
|  | *Fth1^+/+^* | IFN+LPS | 21,78 ± 1,56 | 13,38 ± 1,64 | 16,16 ± 1,62 | 25,78 ± 1,25 | 18,40 ± 1,15 | 28,36 ± 2,26 | 19,355 ± 0,96 |  |  |  |  |  |  |
|  | *Fth1^-/-^* | IFN+LPS | 21,15 ± 0,74 | 17,48 ± 1,21 | 15,25 ± 1,02 | 25,78 ± 1,59 | 18,02 ± 1,12 | 29,27 ± 0,85 | 21,12 ± 1,34 |  |  |  |  |  |  |

^a^ The values are presented as average CT ± SD of at least three independent cultures per condition.
